# Meal timing disruption in maintenance dialysis patients: associations with sleep quality and fatigue

**DOI:** 10.3389/fnut.2026.1815750

**Published:** 2026-05-29

**Authors:** Hong Chen, Lu Xu, Shiqi Ai, Ming Li, Nana Luo, Mingming Wei, Xinjian Li, Weisong Wang

**Affiliations:** 1 Department of Hemopuriffcation, Affiliated Hospital of Jining Medical University, Jining, China; 2 Department of Nephrology, Affiliated Hospital of Jining Medical University, Jining, China; 3 Department of Pain Treatment, Integrative Medicine Hospital of Jining City, Jining, China

**Keywords:** chrononutrition, fatigue, hemodialysis, maintenance dialysis, meal timing, nocturnal eating, peritoneal dialysis, sleep quality

## Abstract

**Background:**

Maintenance dialysis patients frequently experience sleep disturbance and fatigue, which may contribute to disrupted eating timing, yet dialysis-specific evidence on meal timing disruption remains limited.

**Methods:**

We conducted a single-center cross-sectional study among 383 adults receiving maintenance hemodialysis or peritoneal dialysis at Jining Medical University Affiliated Hospital. Sleep quality, fatigue, and depressive symptoms were assessed with the Pittsburgh Sleep Quality Index (PSQI), FACIT-Fatigue (FACIT-F), and PHQ-2. Nocturnal eating was defined as caloric intake at or after 22:00; frequent nocturnal eating (primary outcome) was ≥3 of the past 7 days. We used multivariable logistic regression with progressively adjusted tiered models, indirect association decomposition with bootstrap confidence intervals, and E-value sensitivity analysis. Model 4 (fully adjusted) served as the primary model; all variance inflation factors were below 1.14.

**Results:**

Mean age was 52.9 ± 12.1 years; 55.1% were male and 76.5% received hemodialysis. Mean PSQI was 8.1 ± 3.1 and 85.1% had poor sleep (PSQI >5). Any and frequent nocturnal eating occurred in 54.3% and 15.7%, respectively. In Model 4, higher PSQI was associated with higher odds of frequent nocturnal eating (adjusted OR 1.19 per point, 95% CI 1.07–1.32), while higher FACIT-F (less fatigue) was associated with lower odds (adjusted OR 0.96, 95% CI 0.93–1.00). PHQ-2 positivity was also associated with frequent nocturnal eating (adjusted OR 2.40, 95% CI 1.29–4.47). E-values were 1.66 for PSQI and 4.23 for PHQ-2 positivity, indicating robustness to moderate unmeasured confounding. A descriptive cross-sectional statistical decomposition yielded a fatigue-attributable component of 0.019 log-odds (95% CI −0.000 to 0.045), a modest and non-robust contribution. Sensitivity analyses with alternative outcome cutoffs yielded consistent PSQI and PHQ-2 associations.

**Conclusion:**

Meal timing disruption is common in dialysis and is associated with poorer sleep quality, greater fatigue, and depressive symptoms. Incorporating symptom screening into nutrition assessment may support targeted counseling for meal timing behavior in this population.

## 1 Introduction

Maintenance dialysis sustains life for patients with end-stage kidney disease, yet symptom burden remains high and quality of life is frequently compromised. Beyond biochemical targets, daily functioning in dialysis is shaped by sleep quality, fatigue, appetite, and the feasibility of adhering to complex dietary prescriptions. Sleep complaints are common in both hemodialysis and peritoneal dialysis, and poorer sleep has been linked to worse patient-reported outcomes and adverse clinical profiles in multiple dialysis cohorts (1–3). These observations indicate that sleep disturbance in dialysis is clinically meaningful and may cluster with other symptom domains relevant to nutrition-related self-management.

Nutrition management is central in dialysis care, but nutritional risk persists despite routine counseling. Traditional dialysis nutrition research has focused on nutrient composition, protein-energy wasting, interdialytic weight gain, and mineral metabolism. In parallel, chrononutrition research has highlighted that meal timing and the distribution of intake across the day can influence metabolic physiology through circadian mechanisms (4–6). In non-renal populations, later eating, irregular eating schedules, and night eating patterns have been associated with sleep disturbance, mood symptoms, and adverse cardiometabolic profiles, although causal inference varies across study designs (7, 8). Collectively, this work suggests that when people eat may interact with sleep and symptom states in ways that shape appetite regulation and behavioral routines.

Dialysis patients may be especially susceptible to meal timing disruption for both biological and practical reasons. Chronic kidney disease has been associated with circadian disruption related to metabolic abnormalities, inflammation, symptom patterns, and treatment schedules (9–12). In-center hemodialysis imposes fixed treatment times that can compress daytime eating opportunities, shift meals toward later hours, and generate post-treatment exhaustion or nausea that delays intake. Peritoneal dialysis regimens, particularly automated therapy, can involve nocturnal exchanges and abdominal fullness that may affect sleep continuity and perceived hunger. Dialysis-related symptoms such as pruritus, restless legs symptoms, cramping, and pain can fragment sleep and extend wake time into late hours, increasing opportunity for late-night intake while also impairing next-day appetite (13, 14). In this context, nocturnal eating or late-night snacking may reflect behavioral adaptation to insomnia, treatment-related constraints, or symptom-driven appetite shifts rather than simple dietary preference.

Fatigue is another dominant symptom in dialysis and may be linked to eating timing. Dialysis-related fatigue is common, debilitating, and multifactorial. Intradialytic and post-dialysis fatigue can persist for hours and impair daily activities, including meal preparation and adherence to structured eating schedules (15–17, 19). The etiology of fatigue in dialysis involves multiple converging factors: anemia and erythropoietin deficiency reduce oxygen delivery to skeletal muscle; chronic inflammation and elevated cytokines such as interleukin-6 and tumor necrosis factor-alpha produce sickness-related behaviors including fatigue and anorexia; uremic toxins contribute to metabolic dysregulation and altered neurotransmitter function; sleep disruption and depression further deplete energy reserves; and physical deconditioning from prolonged sedentary time compounds exertional fatigue (14, 16–18). Conceptually, poorer sleep and higher fatigue may co-occur with later and more irregular eating patterns, including reduced morning appetite and increased nocturnal intake. Understanding whether fatigue is statistically associated with the sleep–meal timing relationship is clinically relevant because fatigue and sleep quality are potentially modifiable targets in routine care.

Despite growing interest in chrononutrition, evidence specific to maintenance dialysis remains limited. Most dialysis studies assess sleep quality or fatigue as independent outcomes (1, 17), or focus on dietary adherence and biochemical indicators without detailed characterization of meal timing behaviors (20, 21). As a result, there is limited evidence on whether sleep quality and fatigue are associated with clinically relevant meal timing disruption in maintenance dialysis, and whether fatigue might statistically account for part of any observed association between sleep quality and frequent nocturnal eating. To our knowledge, this is the first study to quantify the prevalence of nocturnal eating and evaluate its multivariable associations with sleep quality and fatigue in a maintenance dialysis population.

To address this gap, we conducted a cross-sectional, questionnaire-based study among adults receiving maintenance hemodialysis or peritoneal dialysis at a tertiary hospital blood purification center. The study aims were to describe the prevalence and patterns of meal timing disruption, with emphasis on nocturnal eating and morning appetite; to evaluate associations between sleep quality and meal timing outcomes while adjusting for demographic, clinical, symptom, and mood-related covariates; and to evaluate whether fatigue statistically accounts for part of the association between sleep quality and frequent nocturnal eating under a cross-sectional analytic framework. We hypothesized that poorer sleep quality would be associated with more frequent nocturnal eating and weaker morning appetite (8, 22), and that greater fatigue would be associated with more frequent nocturnal eating and may account for part of the observed sleep quality to nocturnal eating association in cross-sectional models (14, 18).

We hypothesized that: (1) poorer sleep quality would be associated with more frequent nocturnal eating after multivariable adjustment; (2) greater fatigue would be associated with more frequent nocturnal eating after multivariable adjustment; and (3) fatigue would descriptively account for a modest fraction of the observed sleep quality–nocturnal eating association in cross-sectional models.

This work contributes to dialysis nutrition research in several ways. It operationalizes meal timing disruption as a measurable behavioral phenotype in a high-risk clinical population, integrates sleep quality and fatigue into a unified analytic framework grounded in chrononutrition concepts (5, 6), and provides modality-relevant data that can inform practical counseling. By identifying correlates of nocturnal eating and irregular meal timing, results can support targeted interventions that address symptom management and behavioral strategies alongside traditional nutrient-based counseling, with the longer-term goal of improving nutritional risk profiles and patient-centered outcomes in maintenance dialysis.

## 2 Methods

### 2.1 Study design and setting

This single-center, cross-sectional, questionnaire-based study was conducted in the Blood Purification Center of The Affiliated Hospital of Jining Medical University. The study examined associations of sleep quality and fatigue with meal timing disruption among adults receiving maintenance dialysis. Recruitment and data collection were conducted from February 15, 2024 to September 15, 2025. Each participant completed the questionnaire once during routine dialysis sessions or dialysis outpatient visits.

### 2.2 Participants and recruitment

Adult patients (18 years or older) receiving maintenance hemodialysis or peritoneal dialysis for at least 3 months were consecutively approached during routine dialysis sessions or outpatient visits. Patients were eligible if they were clinically stable and able to complete the questionnaire independently or with trained staff assistance. Exclusion criteria included moderate-to-severe cognitive impairment or severe psychiatric illness precluding valid survey completion, major acute cardiovascular or cerebrovascular events or severe infection in the prior 3 months, concurrent participation in interventional studies likely to affect sleep or eating behaviors, or refusal to participate.

### 2.3 Data collection procedures and clinical data abstraction

Questionnaires were administered by trained staff using standardized instructions. Hemodialysis participants completed surveys during a clinically stable period of the dialysis session or immediately before or after treatment. Peritoneal dialysis participants completed surveys during clinic visits. For participants with reading or writing difficulty, interviewer-assisted administration was permitted; staff read questions verbatim and recorded responses without interpretation. Questionnaires were checked onsite for completeness and logical consistency.

Demographic and dialysis-related information was collected by self-report and verified against medical records when available. Routine laboratory values (albumin, phosphate, potassium, hemoglobin) from a stable period within approximately 1 month of survey completion were abstracted for descriptive purposes and sensitivity analyses when permitted by institutional policy and participant authorization.

### 2.4 Measures

Sleep quality was assessed using the Pittsburgh Sleep Quality Index (PSQI), producing a global score with higher values indicating poorer sleep. Poor sleep was defined as PSQI greater than 5. Fatigue was assessed using the FACIT-Fatigue scale (FACIT-F), with higher scores indicating less fatigue. Depressive symptoms were screened using the Patient Health Questionnaire-2 (PHQ-2), and a positive screen was defined as PHQ-2 of 3 or higher. Dialysis-related symptom burden was quantified using a brief symptom impact module rating the extent to which common dialysis symptoms affected sleep or eating over the past seven days on a 0 to 4 scale, aggregated into a composite symptom burden score.

Meal timing disruption was operationalized using a dialysis-adapted nocturnal eating module. Nocturnal eating was defined as any caloric food or beverage intake occurring at or after 22:00 local time, excluding plain water and non-caloric beverages. Nocturnal eating frequency was measured as the number of days with nocturnal eating during the past seven days. The prespecified primary outcome, frequent nocturnal eating, was defined as nocturnal eating on three or more days in the past seven days. Secondary outcomes included the number of nocturnal eating episodes per night among participants reporting any nocturnal eating, perceived loss of control over nocturnal eating, morning appetite rated on a 1 to 4 ordinal scale, and breakfast skipping frequency captured using ordinal response categories. We acknowledge that the 22:00 cutoff is pragmatic and behavioral in nature; it does not claim to align with a specific metabolic or circadian threshold validated in dialysis populations, but rather serves as an identifiable, patient-reportable marker of late-evening caloric intake. Because no internationally validated, dialysis-specific instrument for nocturnal eating timing currently exists, we interpret this measure as a behavioral indicator rather than a clinical syndrome definition.

### 2.5 Meal timing questionnaire development and psychometric evaluation

Because dialysis-specific meal timing instruments are limited, the nocturnal eating and meal timing module was developed using a structured process. Items were generated from chrononutrition constructs (meal timing, nocturnal intake, appetite rhythm) and dialysis-specific clinical considerations (dialysis schedule, symptom-driven awakenings, dietary restriction burden). Content validity was assessed by a multidisciplinary panel of seven experts (two nephrologists, two renal dietitians, three dialysis nurses). Item-level content validity indices ranged from 0.86 to 1.00, and the scale-level content validity index (S-CVI/Ave) was 0.95 after iterative revision.

A pilot evaluation was conducted in a sample of 40 stable maintenance dialysis patients prior to main enrollment to assess internal consistency and short-term stability. A five-item nocturnal eating severity index (frequency, episodes, timing regularity, perceived control, and sleep-linked eating) demonstrated acceptable internal consistency (Cronbach's alpha 0.83). Two-week test-retest reliability in 25 participants showed good stability (intraclass correlation coefficient 0.86 for the composite score), and weighted kappa for the primary binary classification (frequent nocturnal eating yes or no) was 0.79. Exploratory factor analysis supported a two-factor structure (behavioral frequency and control; circadian timing and appetite) with Kaiser-Meyer-Olkin measure of 0.79 and Bartlett's test *p* less than 0.001, explaining 62 percent of total variance. Psychometric results were used to confirm measurement suitability for group-level analyses, while clinical inference and hypothesis testing relied on the main study regression models using the prespecified primary outcome definition.

### 2.6 Statistical analysis

Descriptive statistics were presented as mean plus or minus standard deviation for approximately normal continuous variables, median with interquartile range for skewed distributions, and count with percentage for categorical variables. Group comparisons between participants with and without frequent nocturnal eating used Welch's *t*-tests for continuous variables, Mann-Whitney U tests for dialysis vintage, and chi-square tests for categorical variables.

#### 2.6.1 Sample size justification

The final analytic sample of 383 participants exceeded the minimum of 10 events per variable recommended for reliable multivariable logistic regression estimation, and satisfied the 20-to-1 rule of thumb preferred in some guidelines. With approximately 60 frequent nocturnal eating events and 8 to 9 covariates across models, the event-per-variable ratio was approximately 7:1 to 8:1, which is commonly considered adequate in epidemiologic research.

#### 2.6.2 Tiered multivariable models

Multivariable logistic regression models estimated adjusted odds ratios and 95 percent confidence intervals for frequent nocturnal eating using four progressively adjusted tiered models. Model 1 included PSQI alone with demographic covariates (age per 10 years, sex, dialysis modality, dialysis vintage per 12 months, body mass index). Model 2 added PHQ-2 positive screen and symptom burden. Model 3 further added FACIT-F. Model 4 (primary model) additionally included diabetes. This tiered approach allowed examination of coefficient stability across nested adjustment sets and served as a form of sensitivity analysis to confounding structure. Model 4 was prespecified as the primary model for inference.

#### 2.6.3 Regression assumptions and model diagnostics

Linearity of the logit for continuous covariates was assessed using the Box-Tidwell approach. The Hosmer-Lemeshow goodness-of-fit test was reported for the primary model. Influential observations were evaluated using Cook's distance. Collinearity among symptom-related measures (PSQI, FACIT-F, PHQ-2, symptom burden) was assessed using variance inflation factors (VIFs). All VIFs were below 1.14, indicating minimal collinearity concern.

#### 2.6.4 Descriptive cross-sectional statistical decomposition

A product-of-coefficients approach with bootstrap confidence intervals (5,000 resamples) was used to decompose the association between PSQI and frequent nocturnal eating into components statistically attributable to FACIT-F under cross-sectional models. Because exposure, intermediate variable, and outcome were measured at one time point, indirect effect estimates were interpreted as a descriptive statistical decomposition without established temporality rather than evidence of a causal pathway.

#### 2.6.5 E-value sensitivity analysis

To quantify sensitivity to unmeasured confounding, E-values were computed for the primary exposure (PSQI) and for PHQ-2 positivity in Model 4. The E-value represents the minimum strength of association that an unmeasured confounder would need to have with both the exposure and the outcome to fully explain away the observed association, conditional on measured covariates. E-values were computed using the methodology of VanderWeele and Ding and reported alongside the observed odds ratios and confidence bounds.

#### 2.6.6 Sensitivity analyses

Sensitivity analyses included: repeating models with PSQI categorized as poor sleep (PSQI > 5); testing a reverse-direction decomposition with FACIT-F specified as the exposure and PSQI specified as the intermediate variable; and repeating primary models using alternative outcome cutoffs for frequent nocturnal eating (at least 1, at least 2, at least 3, and at least 4 days in the past 7 days) to assess whether associations were sensitive to threshold choice.

#### 2.6.7 Unmeasured confounders

We recognize that residual confounding from unmeasured variables (including dialysis shift timing, obstructive sleep apnea and restless legs syndrome, sedative or hypnotic use, residual kidney function, and peritoneal dialysis regimen details) could affect the observed associations. E-values and sensitivity analyses were used to bound the potential influence of such unmeasured confounders.

Two-sided *p* values less than 0.05 were considered statistically significant. Analyses were performed in R (version 4.3.2).

### 2.7 Ethical considerations and informed consent

The study protocol was approved by the ethics committee of The Affiliated Hospital of Jining Medical University (approval number 15701). All participants provided written informed consent prior to participation. Questionnaire responses were de-identified for analysis, and data were stored on password-protected systems accessible only to the study team.

## 3 Results

### 3.1 Participant flow and analytic sample

**Figure 1:**
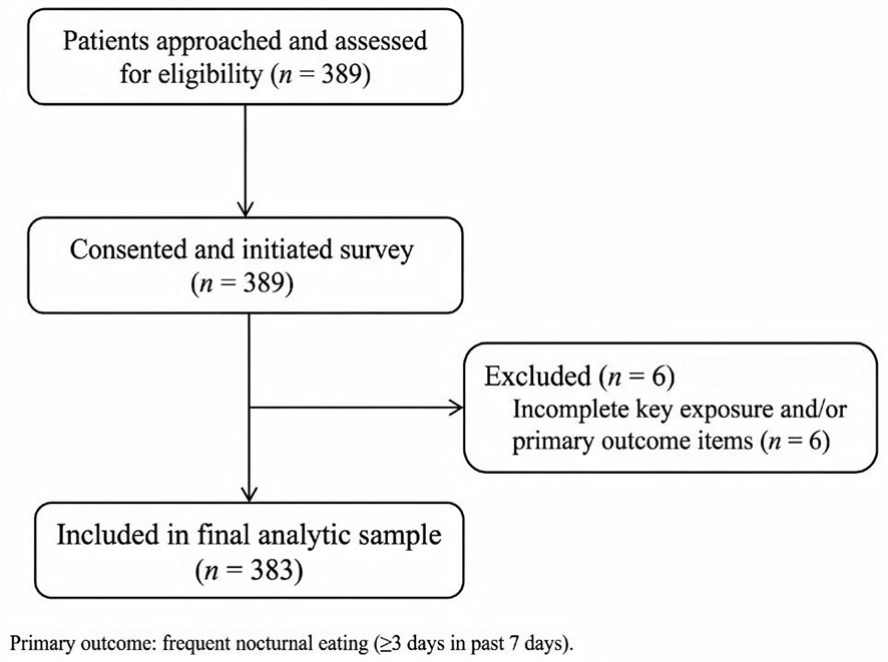
Participant flow and analytic sample. Flow diagram showing recruitment, exclusions due to incomplete exposure and or primary outcome items, and the final analytic sample included in the cross-sectional analysis (*N* = 383).

A total of 389 patients consented and initiated the survey. Six were excluded because the questionnaire was incomplete for the prespecified exposure and or primary outcome items. The final analytic sample included 383 participants (Figure 1).

### 3.2 Baseline characteristics

Participants had a mean age of 52.9 ± 12.1 years, and 55.1% were male. Most received hemodialysis (76.5%), and the median dialysis vintage was 37.6 months (IQR 21.1 to 55.8). Mean body mass index was 23.3 ± 3.7 kg/m^2^. Diabetes was present in 38.1%, and cardiovascular disease in 23.8%. Additional baseline and laboratory characteristics are shown in Table 1.

**Table 1:** Baseline demographic and clinical characteristics (*N* = 383).

Characteristic	Total
Age, years	52.9 ± 12.1
Male sex, *n* (%)	211 (55.1%)
Hemodialysis, *n* (%)	293 (76.5%)
Peritoneal dialysis, *n* (%)	90 (23.5%)
Dialysis vintage, months	37.6 (21.1, 55.8)
BMI, kg/m^2^	23.3 ± 3.7
Diabetes, *n* (%)	146 (38.1%)
Cardiovascular disease, *n* (%)	91 (23.8%)
Education ≥college, *n* (%)	130 (33.9%)
Employed, *n* (%)	75 (19.6%)
Albumin, g/L	37.6 ± 3.6
Phosphate, mmol/L	1.72 ± 0.44
Potassium, mmol/L	4.99 ± 0.50
Hemoglobin, g/L	100 ± 14

### 3.3 E-value sensitivity analysis

E-values for the primary Model 4 associations were 1.66 for PSQI (lower CI bound 1.28) and 4.23 for PHQ-2 positivity (lower CI bound 2.36). The PHQ-2 E-value of 4.23 indicates that an unmeasured confounder would need to be associated with both PHQ-2 positivity and frequent nocturnal eating by a risk ratio of at least 4.23-fold above and beyond measured covariates to fully explain away the observed association, and even more to explain the lower confidence bound. The PSQI E-value of 1.66 suggests moderate robustness: a moderate unmeasured confounder could potentially explain the point estimate, but would need to be stronger than 1.28 to nullify the lower confidence bound. These results suggest the PHQ-2 association is relatively robust to unmeasured confounding, while the PSQI association, though statistically significant, is more sensitive to moderate unmeasured confounding.

### 3.4 Sensitivity analyses across outcome cutoffs

Repeating the primary Model 4 specification across alternative outcome cutoffs for frequent nocturnal eating yielded consistent PSQI and PHQ-2 associations (Supplementary Table). For PSQI, adjusted odds ratios per point were 1.13 (at least 1 day; 95% CI 1.05 to 1.22), 1.13 (at least 2 days; 95% CI 1.04 to 1.23), 1.19 (at least 3 days; primary), and 1.27 (at least 4 days; 95% CI 1.11 to 1.45). PHQ-2 positivity associations were similarly consistent across thresholds (at least 1 day: aOR 2.20; at least 2 days: aOR 2.22; at least 3 days: aOR 2.40; at least 4 days: aOR 2.48). FACIT-F associations were modest and directionally consistent (aORs 0.97 to 0.98 per point) but did not reach conventional statistical significance at all thresholds. These patterns suggest that the PSQI and PHQ-2 associations are not artifacts of the primary cutoff choice.

### 3.5 Sleep quality, fatigue, depressive symptoms, and symptom burden

Sleep disturbance and fatigue were common. The mean PSQI was 8.1 ± 3.1 and 85.1% met the poor-sleep threshold (PSQI >5). The mean FACIT-F score was 25.7 ± 8.5. A total of 29.5% screened positive on PHQ-2 (≥3). Mean symptom burden score was 9.0 ± 4.5. Full distributions are summarized in Table 2.

**Table 2:** Sleep quality, fatigue, depressive symptoms, and symptom burden (*N* = 383).

Measure	Total
PSQI total score	8.1 ± 3.1
Poor sleep (PSQI >5), *n* (%)	326 (85.1%)
FACIT-F total score	25.7 ± 8.5
PHQ-2 total score	2 (1, 3)
PHQ-2 positive screen (≥3), *n* (%)	113 (29.5%)
Symptom burden score	9.0 ± 4.5

### 3.6 Meal timing disruption and nocturnal eating patterns

**Figure 2:**
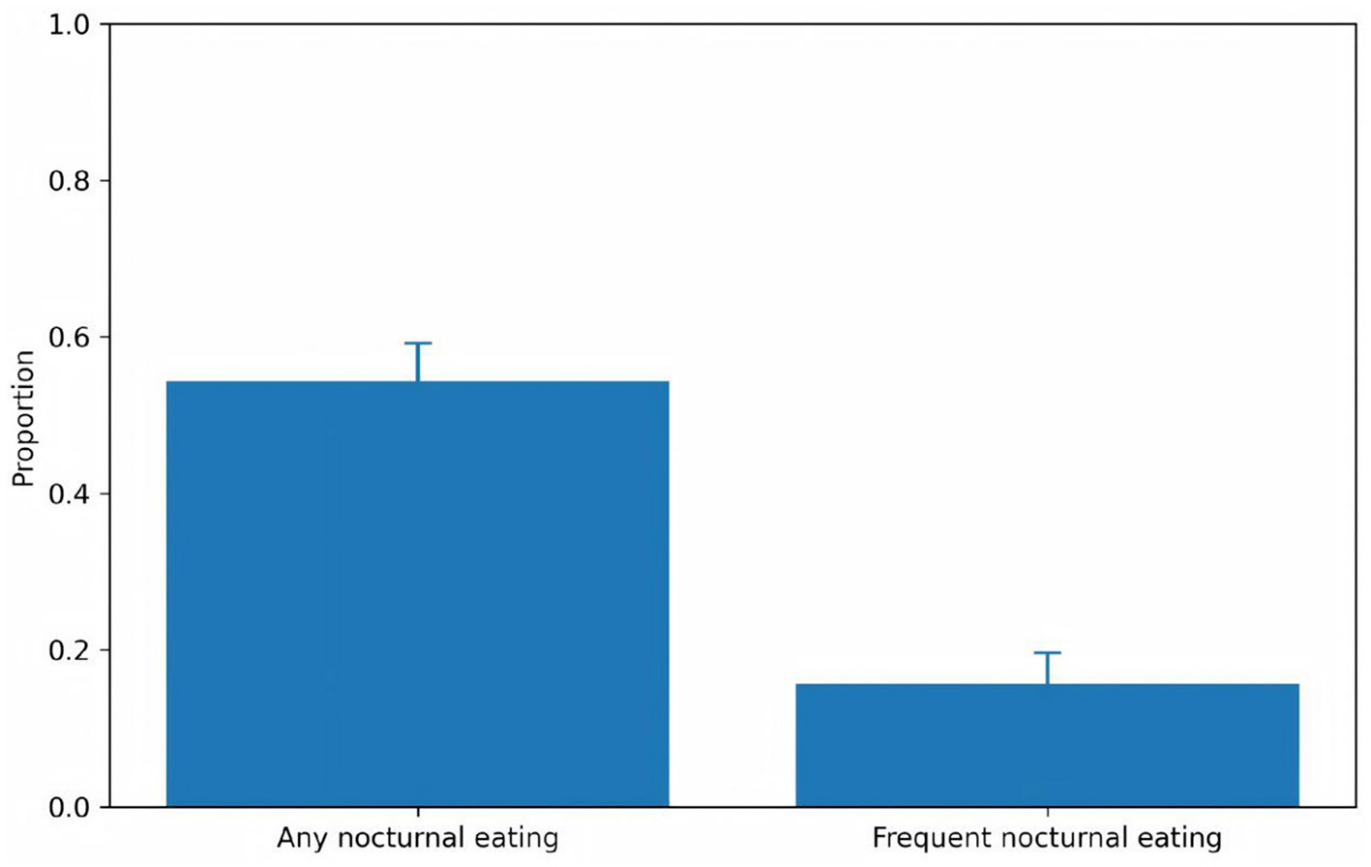
Prevalence of nocturnal eating patterns in maintenance dialysis patients. Bars show the proportion of participants reporting any nocturnal eating (at least 1 day in the past 7 days) and frequent nocturnal eating (3 or more days in the past 7 days). Error bars indicate 95% confidence intervals computed using the Wilson binomial method.

Nocturnal eating was frequently reported. The median number of nocturnal-eating days in the past week was 1 (IQR 0 to 2). Any nocturnal eating (at least 1 day in the past 7 days) occurred in 54.3% of participants, and frequent nocturnal eating (3 or more days in the past 7 days) occurred in 15.7% (Figure 2). Among nocturnal eaters (n = 208), 57.7% reported one episode per nocturnal-eating night, 28.4% reported two episodes, and 13.9% reported three or more. Morning appetite averaged 2.30 ± 0.62 on the 1 to 4 scale. Breakfast skipping was common, with 14.4% reporting never skipping breakfast and 20.4% reporting skipping breakfast at least 5 times per week (Table 3).

**Table 3:** Meal timing disruption and nocturnal eating patterns (*N* = 383).

Variable	Total
Nocturnal eating days in past 7 days	1 (0, 2)
Any nocturnal eating (≥1 day/7 days), *n* (%)	208 (54.3%)
Frequent nocturnal eating (≥3 days/7 days), *n* (%)	60 (15.7%)
Typical nocturnal eating episodes per night, *n* (%), among nocturnal eaters (*n* = 208)	
Once	120 (57.7%)
Twice	59 (28.4%)
≥3 times	29 (13.9%)
Perceived loss of control over nocturnal eating, *n* (%)	
Never	283 (73.9%)
Sometimes	73 (19.1%)
Often	25 (6.5%)
Always	2 (0.5%)
Morning appetite score (1 to 4)	2.30 ± 0.62
Breakfast skipping frequency, *n* (%)	
Never	55 (14.4%)
1 to 2 times per week	134 (35.0%)
3 to 4 times per week	116 (30.3%)
≥5 times per week	78 (20.4%)

### 3.7 Comparisons by frequent nocturnal eating status

**Figure 3:**
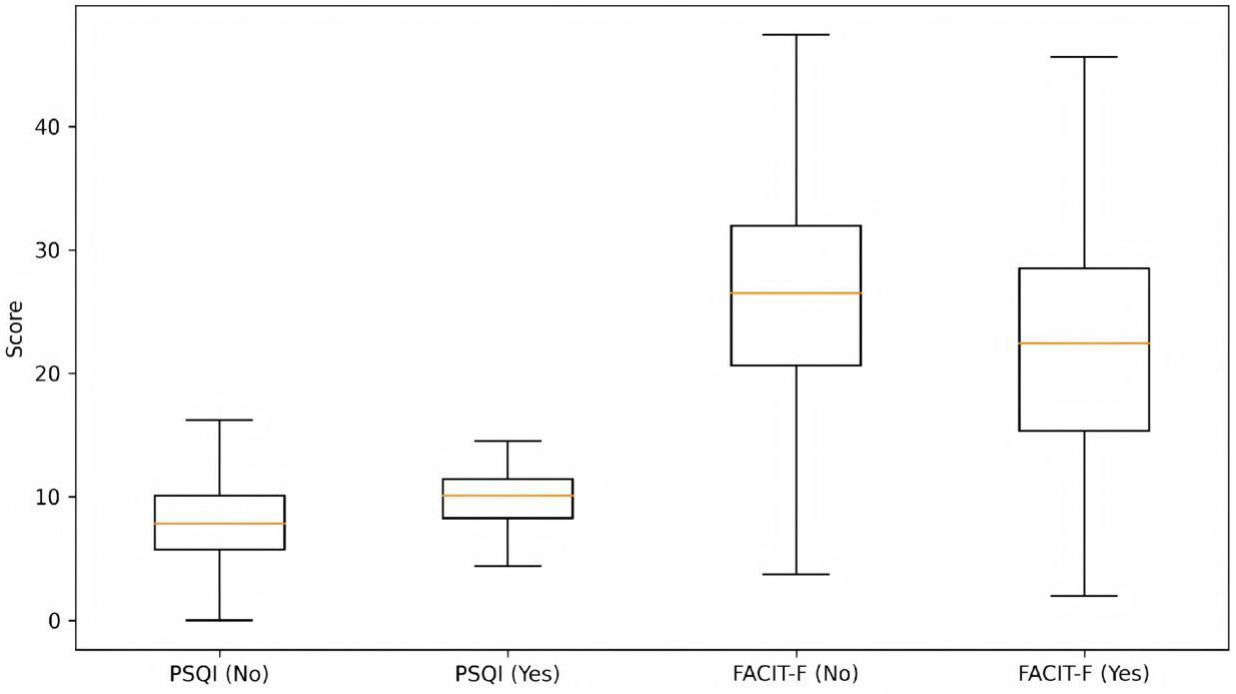
Distributions of sleep quality and fatigue by frequent nocturnal eating status. Boxplots compare PSQI global score and FACIT-F score between participants with and without frequent nocturnal eating. Higher PSQI indicates worse sleep quality; higher FACIT-F indicates less fatigue. Boxes represent the interquartile range with median lines; whiskers indicate dispersion with outliers suppressed for readability.

Participants with frequent nocturnal eating had worse sleep quality and greater fatigue compared with those without frequent nocturnal eating. Mean PSQI was higher in the frequent nocturnal eating group (9.5 ± 3.0) than the non-frequent group (7.9 ± 3.1; *p* < 0.001). FACIT-F was lower (23.5 ± 8.2 vs. 26.1 ± 8.6; *p* = 0.036), indicating greater fatigue (Figure 3). A positive PHQ-2 screen was more common among frequent nocturnal eaters (43.3% vs. 26.9%; *p* = 0.011). Other differences are summarized in Table 4.

**Table 4:** Characteristics by frequent nocturnal eating status (≥3 days/7 days).

Characteristic	Frequent nocturnal eating: yes (*n* = 60)	Frequent nocturnal eating: no (*n* = 323)	*p* value
Age, years	53.6 ± 11.0	52.8 ± 12.3	0.607
Male sex, *n* (%)	28 (46.7%)	183 (56.7%)	0.153
Hemodialysis, *n* (%)	44 (73.3%)	249 (77.1%)	0.529
Dialysis vintage, months	36.8 (21.8, 53.3)	37.8 (20.8, 56.4)	0.94
BMI, kg/m^2^	24.2 ± 3.6	23.2 ± 3.7	0.049
Diabetes, *n* (%)	30 (50.0%)	116 (35.9%)	0.036
PSQI total score	9.5 ± 3.0	7.9 ± 3.1	<0.001
FACIT-F total score	23.5 ± 8.2	26.1 ± 8.6	0.036
PHQ-2 positive screen (≥3), *n* (%)	26 (43.3%)	87 (26.9%)	0.011
Symptom burden score	10.0 ± 4.5	8.8 ± 4.5	0.067

### 3.8 Multivariable associations with frequent nocturnal eating

**Figure 4:**
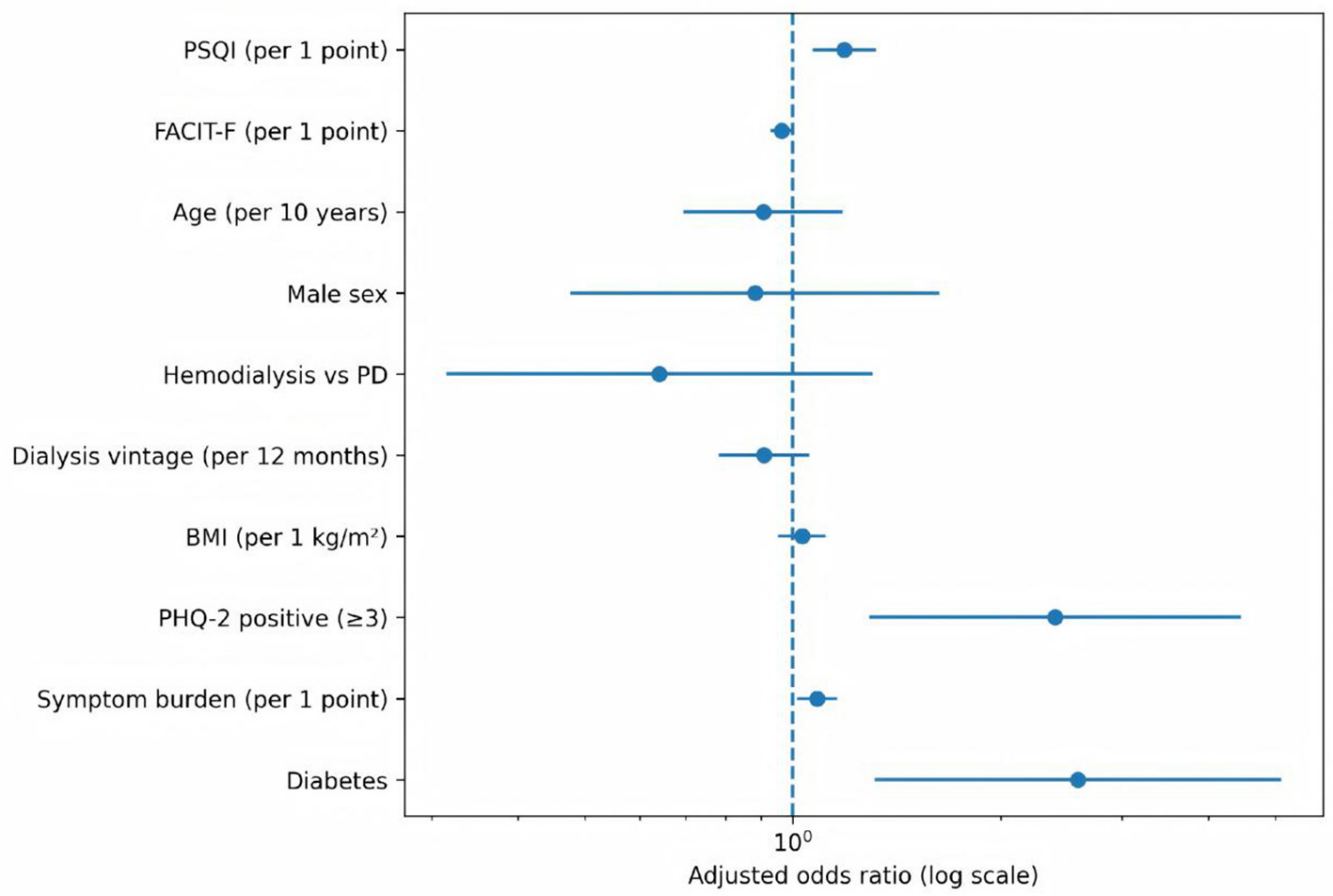
Adjusted associations with frequent nocturnal eating in multivariable logistic regression. Forest plot of adjusted odds ratios (aORs) and 95% confidence intervals from Model 2 for frequent nocturnal eating (3 or more days in the past 7 days). The vertical reference line indicates aOR = 1.0, and the x-axis is on a logarithmic scale. Age is scaled per 10-year increase and dialysis vintage per 12-month increase.

In multivariable logistic regression, poorer sleep quality was independently associated with higher odds of frequent nocturnal eating across all four tiered models (Figure 4; Table 5). In Model 1 (demographic covariates only), each 1-point increase in PSQI was associated with 21% higher odds of frequent nocturnal eating (aOR 1.21, 95% CI 1.09 to 1.34; *p* < 0.001). After adding PHQ-2 and symptom burden in Model 2, the PSQI association was essentially unchanged (aOR 1.21, 95% CI 1.09 to 1.34; *p* < 0.001). After adding FACIT-F in Model 3, PSQI remained significant (aOR 1.19, 95% CI 1.07 to 1.32; *p* = 0.002). In Model 4 (primary model, additionally adjusted for diabetes), PSQI remained statistically significant (aOR 1.19, 95% CI 1.07 to 1.32; *p* = 0.002). Higher FACIT-F (less fatigue) was associated with lower odds of frequent nocturnal eating in Model 4 (aOR 0.96 per point, 95% CI 0.93 to 1.00; *p* = 0.046). A positive PHQ-2 screen was also associated with frequent nocturnal eating (Model 4 aOR 2.40, 95% CI 1.29 to 4.47; *p* = 0.006). Model fit improved across tiers, with AIC values of 301.2 (Model 1), 296.8 (Model 2), 293.1 (Model 3), and 292.7 (Model 4), supporting the inclusion of fatigue and diabetes in the primary model.

**Table 5:** Multivariable logistic regression for frequent nocturnal eating (≥3 days/7 days).

Predictor	Model 1 aOR (95% CI)	*P* value	Model 2 aOR (95% CI)	*p* value
PSQI total score (per 1-point increase)	1.21 (1.09, 1.34)	<0.001	1.19 (1.07, 1.32)	0.002
FACIT-F total score (per 1-point increase)	–	–	0.96 (0.93, 1.00)	0.046
Age (per 10-year increase)	0.92 (0.71, 1.19)	0.525	0.91 (0.70, 1.18)	0.464
Male sex	0.70 (0.40, 1.22)	0.209	0.69 (0.39, 1.22)	0.201
Hemodialysis (vs peritoneal dialysis)	0.92 (0.49, 1.73)	0.789	0.92 (0.48, 1.77)	0.805
Dialysis vintage (per 12-month increase)	1.01 (0.92, 1.10)	0.878	1.01 (0.92, 1.10)	0.889
BMI (per 1 kg/m^2^ increase)	1.07 (1.00, 1.14)	0.055	1.07 (1.00, 1.15)	0.051
PHQ-2 positive screen (≥3)	2.47 (1.35, 4.50)	0.003	2.40 (1.29, 4.47)	0.006
Symptom burden (per 1-point increase)	1.03 (0.98, 1.08)	0.252	1.03 (0.97, 1.08)	0.305
Diabetes	1.69 (0.97, 2.96)	0.064	1.66 (0.94, 2.95)	0.082

### 3.9 Indirect association decomposition involving fatigue

**Figure 5:**
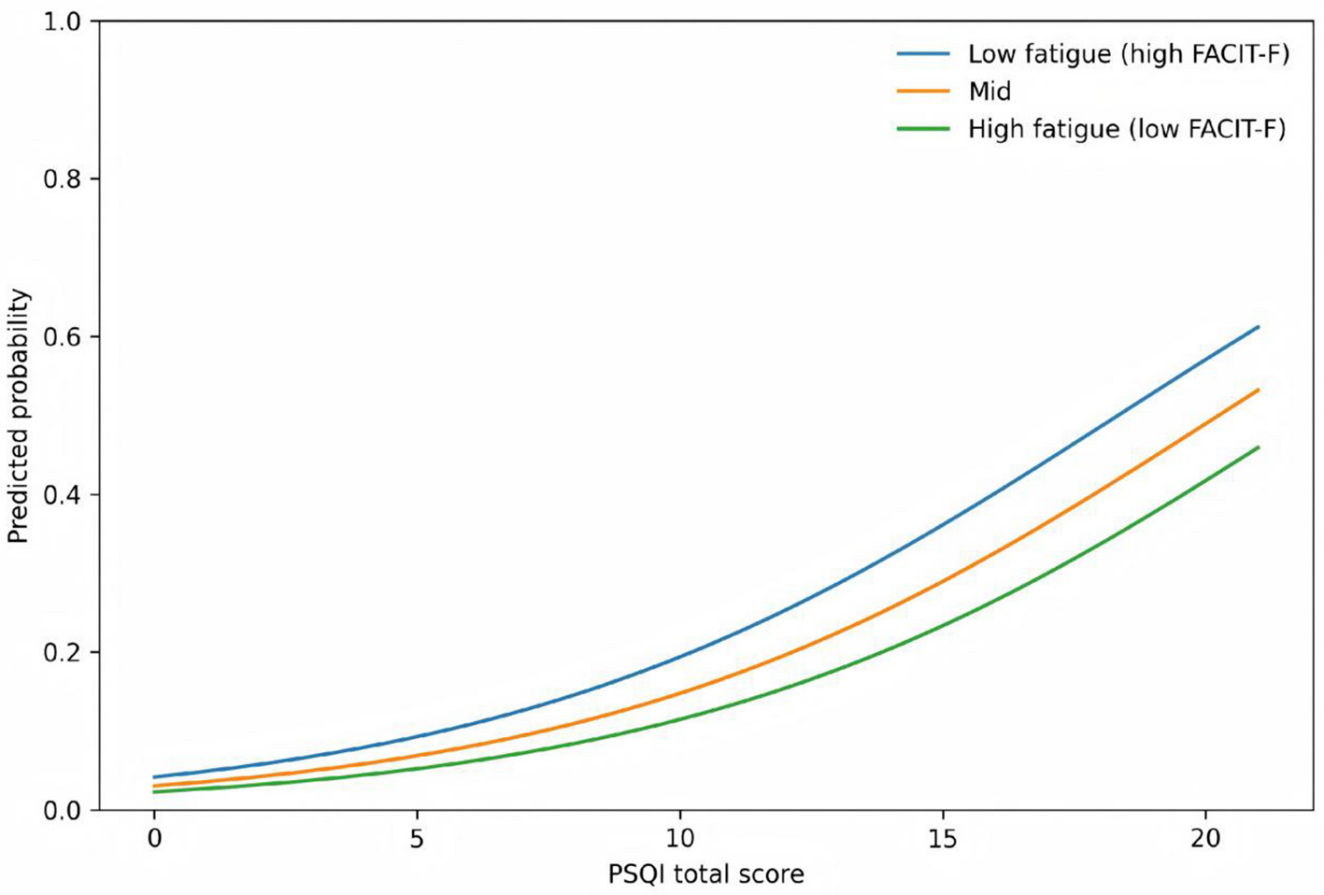
Predicted probability of frequent nocturnal eating across PSQI, stratified by fatigue tertiles. Model-based predicted probability of frequent nocturnal eating across the observed PSQI range, stratified by tertiles of FACIT-F (low fatigue corresponds to higher FACIT-F). Predictions are generated from Model 2 with covariates held at their sample means.

A descriptive cross-sectional statistical decomposition was conducted using bootstrap confidence intervals (5,000 resamples) to partition the association between PSQI and frequent nocturnal eating into components statistically attributable to fatigue (FACIT-F), acknowledging that temporality is not established. The total association component of PSQI with frequent nocturnal eating was 0.189 log-odds per PSQI point (95% CI 0.084 to 0.293; *p* < 0.001). After including FACIT-F, the direct association component was 0.171 log-odds per PSQI point (95% CI 0.065 to 0.276; *p* = 0.002). The fatigue-attributable component was 0.019 log-odds (bootstrap 95% CI −0.000 to 0.045), corresponding to an estimated proportion of 10.0% (bootstrap 95% CI 0.0% to 23.8%). The bootstrap confidence interval touching zero for both the indirect component and proportion indicates this contribution is modest and not robustly distinguishable from zero. These estimates should be interpreted as descriptive partitioning of observed associations rather than causal mediation evidence (Figure 5; Table 6).

**Table 6:** Indirect association decomposition involving fatigue in the association between sleep quality and frequent nocturnal eating.

Effect	Estimate (log-odds)	95% CI	*p* value
Total association component (PSQI → frequent nocturnal eating)	0.189	0.084, 0.293	<0.001
Direct association component (PSQI adjusted for FACIT-F)	0.171	0.065, 0.276	0.002
Indirect association component attributable to FACIT-F	0.019	−0.002, 0.040	0.079
Proportion of association statistically attributable to FACIT-F	0.1	−0.025, 0.226	0.116

## 4 Discussion

In this single-center cross-sectional study of adults receiving maintenance dialysis, meal timing disruption was common and patterned by symptom-related domains. More than half of participants reported nocturnal eating at least once in the prior week, and 15.7% met the prespecified threshold for frequent nocturnal eating, defined as nocturnal eating on three or more days in the past seven days. The behavioral profile suggested that nocturnal eating was not simply an occasional event. Among nocturnal eaters, a substantial proportion reported multiple episodes per night, and breakfast skipping and reduced morning appetite were also frequent. Taken together, the descriptive findings support the concept that disrupted temporal organization of intake may represent a meaningful behavioral phenotype in dialysis, and that characterizing timing behaviors can add information beyond conventional nutrient-centered counseling targets.

The central analytic finding was a robust association between poorer sleep quality and frequent nocturnal eating. In multivariable models adjusting for demographic and dialysis characteristics, depressive symptom screening, symptom burden, and diabetes, each one-point increase in PSQI was associated with higher odds of frequent nocturnal eating. This association persisted after adding fatigue, with partial attenuation rather than disappearance. The direction and magnitude are consistent with chrononutrition and sleep research in non-renal populations, where later eating, irregular timing, and night eating patterns tend to co-occur with insomnia symptoms, delayed sleep timing, or poorer sleep quality (23, 24). In many general-population studies, night eating has been linked to extended wake time and altered appetite regulation, and later eating is often associated with poorer sleep continuity and shorter sleep duration, although causal inference varies (25, 26). Our results extend that pattern to a population with strong schedule constraints and heavy symptom burden, implying that sleep disruption and late-night intake may cluster as part of daily-life adaptation to dialysis and its symptoms rather than reflecting isolated lifestyle preference (27).

Several mechanisms could plausibly explain why sleep disruption and nocturnal eating cluster in dialysis. Fragmented or delayed sleep can lengthen the window of wakefulness, increasing opportunities for intake at night (24). Sleep disruption can also shift hunger and satiety signaling and promote later intake timing, partly through circadian misalignment processes and partly through behavioral drivers such as fatigue, reduced morning appetite, and disrupted routines (25, 28). Dialysis adds multiple layers: treatment schedules can compress daytime eating opportunities, post-treatment symptoms can delay intake, and dialysis-related discomfort such as pruritus, cramps, pain, and restless legs can cause nocturnal awakenings that may prompt snacking (27, 29). At the same time, nocturnal eating itself may worsen sleep through gastric discomfort, reflux, or metabolic arousal, and in dialysis it may be influenced by thirst, dietary restrictions, or glycemic fluctuations in diabetes (23, 30). The cross-sectional nature of our data means a bidirectional or reinforcing loop remains the most defensible interpretation, and the persistence of the PSQI association after accounting for fatigue supports the idea that sleep relates to nocturnal eating through multiple pathways, not solely via fatigue burden (24).

Fatigue was independently associated with frequent nocturnal eating in the fully adjusted model, and fatigue differed by nocturnal eating status in unadjusted comparisons. This aligns with a broader symptom-behavior literature showing that fatigue influences daily routines, physical activity, and self-management capacity (31). In dialysis, fatigue is often described as multifactorial and includes intradialytic and postdialysis components that can persist for hours and shape how patients allocate energy across the day (16, 17). A plausible behavioral pathway is that greater fatigue reduces daytime activity and meal preparation, promotes reliance on convenient foods later in the day, and contributes to irregular eating schedules with delayed first intake and increased evening intake (24). While direct evidence linking fatigue specifically to meal timing is limited in dialysis cohorts, our results help fill that gap by suggesting a measurable relationship between fatigue severity and the timing of intake. Clinically, this matters because fatigue management and sleep optimization are potentially modifiable targets, and improvements in either domain could plausibly support more regular eating timing, even if the direction of effect cannot be confirmed from this design (32).

Mood symptoms were another important correlate. A positive PHQ-2 screen was substantially more common among participants with frequent nocturnal eating and remained strongly associated with frequent nocturnal eating in adjusted models. This finding is consistent with research on night eating syndrome and related behaviors, where depressive symptoms, anxiety, and stress often co-occur with insomnia and late-night intake (7, 8). In chronic disease populations, mood symptoms can influence appetite regulation, perceived control around eating, and adherence to planned routines (31). In dialysis, depressive symptoms have been linked to poorer sleep, greater symptom burden, and lower engagement with self-management behaviors (33–35). Our results support the view that nocturnal eating may be part of a broader symptom network including sleep disruption, fatigue, and mood symptoms rather than a purely nutrition-specific behavior (24, 33, 35). It is also important to interpret PHQ-2 as a screening tool rather than a diagnostic measure. Even so, the association suggests that brief mood screening can provide useful context when clinicians encounter frequent late-night intake, particularly if the behavior is accompanied by insomnia complaints and daytime exhaustion (34, 35).

Unadjusted comparisons also suggested that higher BMI and diabetes were more common among participants with frequent nocturnal eating, and diabetes remained an important clinical covariate in the multivariable models. This pattern aligns with chrononutrition literature linking night eating and later intake timing with higher adiposity and metabolic dysregulation (25, 26, 36). In the general population, later eating and circadian misalignment have been associated with impaired glucose tolerance and adverse cardiometabolic profiles, though effect sizes vary and confounding is common (4, 26, 28). In dialysis, diabetes introduces additional complexity. Glycemic variability, dietary restrictions, and medication timing may influence hunger and late-night intake, and fear of nocturnal hypoglycemia may also prompt nighttime eating in some patients (28, 30). Our analysis treated diabetes as a confounder and clinical marker rather than a primary mechanistic variable, but the observed differences suggest diabetes could be explored as a potential effect modifier in future work, particularly in larger samples that allow stratified modeling. This is also clinically relevant because diabetes is common in dialysis, and counseling on meal timing may need to be tailored to glycemic management strategies (4).

The indirect association decomposition suggests a nuanced story. Fatigue statistically attenuated the association between PSQI and frequent nocturnal eating, but the indirect component was modest and not robust by conventional thresholds. Interpreted conservatively, this indicates that fatigue may explain a limited fraction of the observed sleep to nocturnal eating association, while a larger portion reflects either direct links between sleep disruption and eating timing or shared upstream determinants not captured by the model. This pattern is consistent with dialysis symptom research where sleep, fatigue, mood, and symptom burden are intercorrelated and may share common drivers such as inflammation, anemia, uremic toxicity, and treatment-related stressors (27, 33, 35). It is also consistent with the possibility that nocturnal eating itself contributes to fatigue and poor sleep, which would weaken any attempt to place fatigue strictly “between” sleep and eating (23, 24).

We addressed potential collinearity among symptom measures by reporting variance inflation factors, which were all below 1.14. This indicates that the sleep quality, fatigue, depressive symptom, and symptom burden measures, while conceptually interrelated, do not exhibit problematic multicollinearity in the regression context. The low VIFs support the interpretability of individual coefficient estimates in the tiered models.

We quantified sensitivity to unmeasured confounding using E-values. The PHQ-2 association (E-value = 4.23) is robust to considerable unmeasured confounding, meaning an unmeasured confounder would need to be quite strongly associated with both the exposure and outcome to fully explain away the observed association. The PSQI association (E-value = 1.66) could be explained away by a moderate unmeasured confounder, suggesting that while the association is statistically significant after measured covariate adjustment, it should be interpreted with appropriate caution regarding residual confounding.

### 4.1 Limitations and implications

Several limitations warrant emphasis. The cross-sectional design precludes establishing temporality among sleep quality, fatigue, mood symptoms, and nocturnal eating, so causal interpretations are not justified and bidirectional relationships remain plausible (24). All primary measures were self-reported, which introduces recall and social desirability bias, and the nocturnal eating definition relied on a clock-time threshold and a 7-day recall window without time-stamped dietary logs or objective sleep assessment (23, 24). The study was single-center with predominance of hemodialysis, which may limit generalizability to centers with different schedules and counseling practices. Even with prespecified covariate adjustment, residual confounding is likely from unmeasured factors including dialysis shift timing (morning vs. evening sessions), obstructive sleep apnea and restless legs syndrome, sedative or hypnotic medication use, residual kidney function, and peritoneal dialysis regimen details, and the statistical decomposition should be interpreted as descriptive partitioning of associations rather than causal mediation (33). Despite limitations, the findings have practical implications: when frequent nocturnal eating is identified, clinicians should consider concurrent sleep disruption, fatigue, and mood symptoms, and counseling may be more effective when it integrates symptom management, routine stabilization, and structured plans for evening intake rather than focusing only on nutrient targets (27, 31).

## 5 Conclusions

Meal timing disruption is common, with nocturnal eating reported by more than half of participants and frequent nocturnal eating present in 15.7%. Poorer sleep quality is independently associated with higher odds of frequent nocturnal eating after adjustment for demographic and clinical factors, dialysis characteristics, symptom burden, depressive symptom screening, and diabetes. This association persists with partial attenuation after accounting for fatigue. Greater fatigue and a positive depression screen are also associated with frequent nocturnal eating, supporting the concept that late-night intake in dialysis is embedded within a broader symptom network involving sleep, fatigue, and mood. A descriptive cross-sectional decomposition suggested that fatigue accounted for only a modest fraction of the observed sleep to nocturnal eating association, reinforcing the likelihood of multifactorial and potentially bidirectional relationships. Incorporating brief screening for sleep quality, fatigue, mood symptoms, and late-night intake into routine nutrition assessment may help identify patients at risk for irregular intake timing and support more targeted, symptom-informed counseling. Longitudinal studies with repeated assessments and more objective measures of sleep and dietary timing are needed to determine temporal ordering and to test whether improving sleep and fatigue can reduce nocturnal eating and improve patient-centered outcomes.

## Data Availability

The raw data supporting the conclusions of this article will be made available by the authors, without undue reservation.
